# Supplementary material for: Combined glycerol and sodium bicarbonate elicits improvements in fluid retention and blood buffering capacity
Source: PLoS One. 2026 Feb 4;21(2):e0341245. doi: 10.1371/journal.pone.0341245 (PMC12872017; doi:10.1371/journal.pone.0341245)
Supplement: S1 Table — (DOCX) [file pone.0341245.s001.docx]

|  |  | **CON** | **G** | **SB** | **G+SB** | **Friedman’s p-value** |
| --- | --- | --- | --- | --- | --- | --- |
| **Mean total severity and range of individual participant total severity^a^** | | | | | | |
|  | Overall gut discomfort | 7.3 (0-35) | 4.0 (0-29) | 3.9 (0-16) | 4.5 (0-26) | 0.200 |
|  | **Upper GI Symptoms** | 21.7 (0-94) | 9.9 (0-83) | 5.0 (0-16) | 6.0 (0-40) | 0.797 |
|  | Belching | 1.4 (0-13) | 0.9 (0-9) | 0.8 (0-9) | 0.5 (0-3) | 0.900 |
|  | Heartburn | 0.5 (0-4) | 0.3 (0-3) | 0.2 (0-2) | 0 (0-0) | 0.603 |
|  | Bloating (stomach fullness) | 10.4 (0-46) | 4.3 (0-35) | 1.9 (0-7) | 2.5 (0-14) | 0.212 |
|  | Stomach Pain | 7.0 (0-48) | 4.1 (0-39) | 1.6 (0-11) | 3.1 (0-25) | 0.850 |
|  | Urge to regurgitate | 2.5 (0-21) | 0.4 (0-4) | 0 (0-0) | 0 (0-0) | 0.119 |
|  | Regurgitation | 0 (0-0) | 0 (0-0) | 0.5 (0-5) | 0 (0-0) | 0.392 |
|  | Projectile vomiting | 0 (0-0) | 0 (0-0) | 0 (0-0) | 0 (0-0) | 1.000 |
|  | **Lower GI symptoms** | 5.4 (0-22) | 5.2 (0-20) | 2.5 (0-12) | 2.7 (0-16) | 0.353 |
|  | Flatulence | 0 (0-0) | 0 (0-0) | 0.1 (0-1) | 0.2 (0-2) | 0.706 |
|  | Lower abdominal bloating (abdominal pressure) | 4.5 (0-13) | 1.7 (0-10) | 0.1 (0-1) | 0.5 (0-5) | 0.052 |
|  | Urge to defecate | 0 (0-0) | 0 (0-0) | 0.5 (0-3) | 0.3 (0-2) | 0.112 |
|  | Left intestinal pain | 0 (0-0) | 0 (0-0) | 0 (0-0) | 0 (0-0) | 1.000 |
|  | Right intestinal pain | 0 (0-0) | 0 (0-0) | 0 (0-0) | 0 (0-0) | 1.000 |
|  | Defecation – Normal Consistency | 0 (0-0) | 0.9 (0-10) | 0.9 (0-10) | 1.8 (0-10) | 0.494 |
|  | Defecation: abnormal loose stools consistency | 0.9 (0-10) | 1.8 (0-20) | 0.9 (0-10) | 0 (0-0) | 0.392 |
|  | Defecation: diarrhea | 0 (0-0) | 0 (0-0) | 0 (0-0) | 0 (0-0) | 1.000 |
|  | Defecation: blood stools | 0 (0-0) | 0 (0-0) | 0 (0-0) | 0 (0-0) | 1.000 |
|  | **Other GI symptoms** | 8.6 (0-71) | 8.4 (0-71) | 5.5 (0-27) | 12.8 (0-80) | 0.602 |
|  | Nausea | 4.5 (0-26) | 4.5 (0-28) | 2.3 (0-12) | 5.6 (0-31) | 0.697 |
|  | Dizziness | 1.9 (0-21) | 2.0 (0-22) | 3.0 (0-18) | 5.1 (0-26) | 0.082 |
|  | Stitch | 2.2 (0-24) | 1.9 (0-21) | 0.3 (0-3) | 2.1 (0-23) | 0.392 |
|  | **Total GI symptoms^d^** | 35.7 (0-177) | 23.5 (0-104) | 13.0 (0-46) | 20.6 (0-98) | 0.435 |
| **Incidence (%) of GI symptoms (rating ≥1 at any time point)^b^** | | | | | |  |
|  | Overall gut discomfort | NA | NA | NA | NA | NA |
|  | **Upper GI symptoms** | 53.6 | 36.4 | 81.8 | 63.6 | NA |
|  | Belching | 18.2 | 18.2 | 9.1 | 27.3 | NA |
|  | Heartburn | 18.2 | 9.2 | 9.1 | 0 | NA |
|  | Bloating (stomach fullness) | 54.5 | 27.3 | 45.5 | 36.4 | NA |
|  | Stomach Pain | 27.6 | 27.3 | 36.4 | 36.4 | NA |
|  | Urge to regurgitate | 27.6 | 9.1 | 0 | 0 | NA |
|  | Regurgitation | 0 | 0 | 9.1 | 0 | NA |
|  | Projectile vomiting | 0 | 0 | 0 | 0 | NA |
|  | **Lower GI symptoms** | 45.5 | 45.5 | 36.4 | 36.4 | NA |
|  | Flatulence | 0 | 9.1 | 9.1 | 9.1 | NA |
|  | Lower abdominal bloating (abdominal pressure) | 45.5 | 18.2 | 9.1 | 9.1 | NA |
|  | Urge to defecate | 0 | 0 | 18.2 | 18.2 | NA |
|  | Left intestinal pain | 0 | 0 | 0 | 0 | NA |
|  | Right intestinal pain | 0 | 0 | 0 | 0 | NA |
|  | Defecation – Normal Consistency | 0 | 9.1 | 9.1 | 18.2 | NA |
|  | Defecation: abnormal loose stools consistency | 9.1 | 19.2 | 9.1 | 0 | NA |
|  | Defecation: diarrhea | 0 | 0 | 0 | 0 | NA |
|  | Defecation: blood stools | 0 | 0 | 0 | 0 | NA |
|  | **Other GI symptoms** | 36.4 | 36.4 | 36.4 | 36.4 | NA |
|  | Nausea | 36.4 | 36.4 | 27.3 | 36.4 | NA |
|  | Dizziness | 9.1 | 9.1 | 27.3 | 36.4 | NA |
|  | Stitch | 9.1 | 9.1 | 9.1 | 9.1 | NA |
|  | **Total GI symptoms** | 72.7 | 63.6 | 100 | 81.8 | NA |
| **Incidence (%) of severe GI symptoms (rating ≥5 at any time point)^c^** | | | | | | |
|  | Overall gut discomfort | NA | NA | NA | NA | NA |
|  | **Upper GI symptoms** | 45.5 | 18.2 | 18.2 | 9.1 | NA |
|  | Belching | 0 | 0 | 0 | 0 | NA |
|  | Heartburn | 0 | 0 | 0 | 0 | NA |
|  | Bloating (stomach fullness) | 36.4 | 9.1 | 0 | 0 | NA |
|  | Stomach Pain | 18.2 | 9.1 | 0 | 9.1 | NA |
|  | Urge to regurgitate | 9.1 | 0 | 0 | 0 | NA |
|  | Regurgitation | 0 | 0 | 9.1 | 0 | NA |
|  | Projectile vomiting | 0 | 0 | 0 | 0 | NA |
|  | **Lower GI symptoms** | 27.3 | 18.2 | 18.2 | 18.2 | NA |
|  | Flatulence | 0 | 0 | 0 | 0 | NA |
|  | Lower abdominal bloating (abdominal pressure) | 18.2 | 0 | 0 | 0 | NA |
|  | Urge to defecate | 0 | 0 | 0 | 0 | NA |
|  | Left intestinal pain | 0 | 0 | 0 | 0 | NA |
|  | Right intestinal pain | 0 | 0 | 0 | 0 | NA |
|  | Defecation – Normal Consistency | 0 | 9.1 | 9.1 | 18.2 | NA |
|  | Defecation: abnormal loose stools consistency | 9.1 | 9.1 | 9.1 | 0 | NA |
|  | Defecation: diarrhea | 0 | 0 | 0 | 0 | NA |
|  | Defecation: blood stools | 0 | 0 | 0 | 0 | NA |
|  | **Other GI symptoms** | 18.2 | 9.1 | 18.2 | 27.3 | NA |
|  | Nausea | 9.1 | 9.1 | 0 | 9.1 | NA |
|  | Dizziness | 0 | 9.1 | 9.1 | 18.2 | NA |
|  | Stitch | 9.1 | 9.1 | 0 | 0 | NA |
|  | **Total GI symptoms** | 45.5 | 45.5 | 36.4 | 27.3 | NA |

*^a^Total severity values are the sum of all reported GI symptoms at all measured time points from the relevant session. For total severity of all individual GI symptoms, the maximum possible value was 100 (each symptom reported 10 times, with a maximum rating of 10). For total severity for upper, lower, and other GI symptoms, the maximum possible value was 700 (7 symptoms, reported 10 times, each with a maximum rating of 10), 900 (9 symptoms, reported 10 times, each with a maximum rating of 10), and 300 (3 symptoms, reported 10 times, each with a maximum rating of 10) respectively. For total severity for total GI symptoms, the maximum possible value was 1900 (19 symptoms, reported 10 times, each with a maximum rating of 10).*

*^b^Incidence (%) of GI symptoms indicates the percentage of participants which reported a rating ≥1 for the corresponding GI symptom and was calculated as: (number of participants who rated ≥1 for at least one symptom/sample size of 11) × 100).*

*^c^Incidence (%) of severe GI symptoms indicates the percentage of participants which reported a rating ≥5 for the corresponding GI symptom and was calculated as: (number of participants who rated ≥5 for at least one symptom/sample size of 11) × 100).*

*^d^Total GI symptoms summarise upper, lower and other GI symptoms. Upper GI symptoms: belching, heartburn, bloating, stomach pain, urge to regurgitate, regurgitation, and projectile vomiting, Lower GI symptoms: flatulence, lower abdominal bloating, urge to defecate, left and right intestinal pain, and defecation. Other GI symptoms: nausea and dizziness.*

*NA = not applicable. GI = Gastrointestinal.*
