# Supplementary material for: Combined glycerol and sodium bicarbonate elicits improvements in fluid retention and blood buffering capacity
Source: PLoS One. 2026 Feb 4;21(2):e0341245. doi: 10.1371/journal.pone.0341245 (PMC12872017; doi:10.1371/journal.pone.0341245)
Supplement: Attachment — Submitted filename: Response to Reviewers - Dec 2025.docx [file pone.0341245.s003.docx]

**Academic Editor’s Comments to Author:**

1. Please ensure that your manuscript meets PLOS ONE's style requirements, including those for file naming. The PLOS ONE style templates can be found at

<https://journals.plos.org/plosone/s/file?id=wjVg/PLOSOne_formatting_sample_main_body.pdf> and

<https://journals.plos.org/plosone/s/file?id=ba62/PLOSOne_formatting_sample_title_authors_affiliations.pdf>

Many thanks for these resources. We have now adjusted the formatting of our manuscript to the PLOS ONE formatting requirements. We have made the following changes:

- We have adjusted all level 1 headings to bold type, 18pt font, and all level 2 headings to bold type, 16pt font.
- We have adjusted headings to be written in sentence case
- We have adjusted all figure and table titles to use bold type font.
- We have adjusted gastrointestinal symptom tables to include correctly formatted table footnotes

2. When completing the data availability statement of the submission form, you indicated that you will make your data available on acceptance. We strongly recommend all authors decide on a data sharing plan before acceptance, as the process can be lengthy and hold up publication timelines. Please note that, though access restrictions are acceptable now, your entire data will need to be made freely accessible if your manuscript is accepted for publication. This policy applies to all data except where public deposition would breach compliance with the protocol approved by your research ethics board. If you are unable to adhere to our open data policy, please kindly revise your statement to explain your reasoning and we will seek the editor's input on an exemption. Please be assured that, once you have provided your new statement, the assessment of your exemption will not hold up the peer review process.

Thank you for these clarifications. This data is now available through Deakin University’s research repository at the following DOI: <https://doi.org/10.26187/deakin.29434217.v1>

3. Please include captions for your Supporting Information files at the end of your manuscript, and update any in-text citations to match accordingly. Please see our Supporting Information guidelines for more information: <http://journals.plos.org/plosone/s/supporting-information>.

Captions for all supporting information (figures, tables and supplementary tables) have now been provided within lines 696-729 of the revised manuscript.

4. If the reviewer comments include a recommendation to cite specific previously published works, please review and evaluate these publications to determine whether they are relevant and should be cited. There is no requirement to cite these works unless the editor has indicated otherwise.

Thank you for reminding us of this requirement. We have checked all reviewer comments and can confirm that there were no specific works suggested.

5. Please review your reference list to ensure that it is complete and correct. If you have cited papers that have been retracted, please include the rationale for doing so in the manuscript text, or remove these references and replace them with relevant current references. Any changes to the reference list should be mentioned in the rebuttal letter that accompanies your revised manuscript. If you need to cite a retracted article, indicate the article’s retracted status in the References list and also include a citation and full reference for the retraction notice.

Thankyou for this reminder. These checks have now been completed.

**Reviewers' Comments to Author:**

**Reviewer #1**

**General Comments:**

This manuscript addresses an important and timely question regarding hyperhydration strategies in sport and occupational contexts, specifically the combined ingestion of glycerol and sodium bicarbonate. To my knowledge, this is the first study to directly examine the effects of this combination on fluid retention and blood buffering capacity, and the findings offer valuable practical insights for athletes competing in hot environments and situations where pre-event preparation time is limited. The study is well-designed, with a randomized crossover approach, clear methodology, and careful monitoring of hydration, buffering markers, and gastrointestinal symptoms. The results are robust, showing that both glycerol and sodium bicarbonate enhance fluid retention compared to control, and that the combination accelerates retention and enhances buffering capacity without worsening GI symptoms. These findings make a meaningful contribution to the literature on hydration strategies and performance support. Overall, I find the study scientifically sound, ethically conducted, and appropriately reported. With minor adjustments to data availability and interpretation, this manuscript will make a valuable addition to the literature and I recommend it for publication.

Thank you for the time and effort you have spent reviewing our manuscript, it is very much appreciated.

We have provided a response to each of your specific comments and have indicated with text highlighted yellow where we have made changes within the revised manuscript in response.

We appreciate your contributions to our manuscript and feel that your suggestions have improved the revised document.

**Specific Comments:**

1) Your study combines glycerol with sodium bicarbonate (NaHCO₃) for hyperhydration, which you note has not been directly investigated before. Could you more explicitly highlight what new insights this combination provides compared to the well-studied glycerol + NaCl strategy?

Thank you for this suggestion. We have now more explicitly highlighted the new insights that our investigation of the combination of glycerol with sodium bicarbonate provides compared to the well-studied glycerol + NaCl strategy.

The updated text within the revised manuscript reads:

‘Currently the only combined Na^+^ and glycerol hyperhydration strategies to be fully investigated have utilised NaCl (11). However, NaHCO_3_ has recently demonstrated as a source of Na+ to induce hyperhydration (15). Consequently, combining NaHCO_3_ and glycerol may induce additive fluid retention, similar to that previously reported in combined NaCl and glycerol hyperhydration strategies, while also concurrently improving buffering capacity.’

This change appears on lines 93-96 of the revised manuscript.

2) How does your finding that G+SB accelerates fluid retention (but does not enhance peak retention compared to SB or G alone) advance the field in practical terms? For example, could this be positioned as a novel solution for athletes with limited pre-competition preparation time?

Thank you for raising this point. We have now made a change to the revised manuscript to more clearly explain the practical applications of our findings. The updated text reads:

‘A potential practical application of our findings is in the time period immediately before competition, which is often short on the day of major events for endurance competitions. For instance, early morning starts are common for open water swimming, triathlon, marathon/ultra marathon and race-walking events, making G+SB a potentially more feasible hyperhydration strategy than SB or G for athletes competing in these events.’

This change appears on lines 496-501 of the revised manuscript.

3) Could you clarify whether the novelty of your study lies primarily in the physiological insight (timing and buffering interactions) or in the applied context (sport, occupational use), and how this differs from prior work?

Thank you for raising this question. The novelty of our study lies primarily in physiological insight, given that there is no exercise test included in the current study. This study is the first to investigate combined glycerol and NaHCO_3_ hyperhydration and the effects on hydration status, buffering capacity and gastrointestinal symptoms. To add further clarity regarding the novelty of our study and how this differs from prior work, we have added the following text to the updated manuscript:

‘This is the first study to our knowledge to investigate the combined hyperhydration potential of glycerol and NaHCO_3_. The novelty of this study lies primarily in the key physiological insights gained into the timing responses of hydration status, blood buffering capacity, and GI symptoms following combined glycerol and NaHCO_3_ hyperhydration. Future research will be able to utilise the timing-specific insights gathered from our study and integrate exercise testing.’

This change appears on lines 359-363 of the revised manuscript.

4) The study reports no changes in plasma volume despite significant differences in fluid retention. Could you elaborate on why plasma volume may not have reflected fluid retention differences, and whether methodological sensitivity may be a factor?

Thank you for this comment. This outcome could be related to different analysers being used for Hb results in the current investigation and previous investigations, even though valid methodologies were used in each instance. To elaborate further as to how these differences may have resulted in plasma volume not reflecting fluid retention differences, the following section at lines 421-431 has been added to the revised manuscript:

‘Some previous studies have reported (as with the current investigation) changes in some but not all hydration measures (13, 15). One potential explanation for differences between the current investigation and Siegler et al., who reported a significant change in PV, but no significant change in BM, could be due to slight differences in Hb results produced by different analysers, despite both studies using valid instrumentation (ABL800 Flex, Radiometer and i-STAT 1 Wireless, Abbott, respectively) (15). Additionally, in other previous studies where an increase in ΔPV was reported (6, 11), a different analyser (HemoPoint H2, EKF Diagnostics) than that used in the current study was used to generate Hb results. Therefore, future research needs to more thoroughly investigate and compare the results of different analysis methods that can be used to determine Hb.’

5) You used a glycerol dose (1.0 g·kg^-1^ BM) that is lower than some prior studies. Could you justify this choice more explicitly in terms of expected efficacy versus GI tolerance, and whether higher doses might have altered the outcomes?

Thank you for this suggestion. Isolated sodium bicarbonate and glycerol ingestion have been reported to increase the incidence and severity of GI symptoms, therefore a lower dosage of glycerol of 1.0 g·kg^-1^ BM (a dose which has been demonstrated to elicit improvements in fluid retention in previous research), was used to minimise any potential additive effect combined ingestion could have on GI symptoms. To highlight the justification for the glycerol dosage used, the following has been added to the revised manuscript:

‘Given that each supplement can independently increase the incidence and severity of GI symptoms, a lower glycerol dosage of 1.0 g·kg^-1^ BM than most frequently reported in previous studies was utilised during combined supplementation (10, 21, 22). A dose of 1.0 g·kg^-1^ BM glycerol was used as it has been demonstrated to significantly increase fluid retention compared to a fluid bolus ingested alone (22).’

These changes appear in lines 196-201 of the revised manuscript.

To outline how the 1.0 g·kg⁻^1^ BM glycerol dose may have altered the outcome of results, the following has been added to lines 406-412 of the revised manuscript:

‘The combined ingestion of 0.3 1.0 g·kg⁻^1^ BM NaHCO_3_ and 1.0 g·kg^-1^ BM glycerol elicited minimal GI symptoms in the current study, therefore a higher glycerol dosage (~1.2 g·kg⁻^1^ BM) should also be investigated in future research. This specific combination with the glycerol provided at a higher dose may induce increased fluid retention in glycerol treatments compared with that observed in the current investigation, although the combination needs to be specifically tested as it may also elicit an increased incidence and severity of GI symptoms.’

6) Since no additive benefit was observed in peak fluid retention for G+SB compared to single agents, do you think the practical advantage of G+SB is mainly about timing of onset? If so, would you recommend it over SB alone in competitive practice?

Thank you for this suggestion. This is a valuable point which we agree with and have added more clarity surrounding our suggested use of the SB strategy in our practical application section. We have specified that we recommend SB as a suggested strategy alongside G+SB, when concurrent hydration and buffering is required. The following revised text appears on line 501-506 of the revised manuscript:

‘The improvements in buffering capacity observed in this study suggest that G+SB and SB may be beneficial in some specific competitive events where adequate hydration status and buffering capacity may be beneficial (e.g. in the context of sprint finishes in road cycling events which also require prolonged effort (59)), or occupational contexts (e.g. shipboard firefighting performed by Navy personnel (60)).

7) You note that GI symptoms were minimal across conditions. Could you provide more context on whether this is consistent with prior SB-only studies, where GI issues are often reported? What might explain the reduced severity in your cohort?

We thank the reviewer for this comment. A comparison of the GI symptoms results from the current investigation with previous SB-only studies has now been added to the revised manuscript.

‘The minimal GI symptoms reported in the current study may be at least partially explained by participants coingesting NaHCO_3_ with a carbohydrate meal, a strategy reported to reduce the incidence of GI symptoms in comparison to when NaHCO_3_ is ingested without a carbohydrate meal (34). Early studies which reported GI symptoms typically used different types of ingestion protocols, such as those with NaHCO_3_ prepared in a solution and without a coingested carbohydrate meal (54). Over the past two decades however, as the practice of ingesting NaHCO_3_ via capsules and with a carbohydrate meal has been more frequently utilised, NaHCO_3_ studies have more commonly reported low incidence and severity GI symptoms (55).’

This change appears on lines 463-472 of the revised manuscript.

**Reviewer #2:**

**General Comments:**

In this study, investigators sought to assess the potential additive effects of sodium bicarbonate and glycerol on fluid retention and blood buffering parameters in physically active adults. Overall, fluid retention was increased with glycerol and sodium bicarbonate from 120-180 minutes post ingestion compared to control, promoting a slightly earlier time course for fluid retention compared to sodium bicarbonate and glycerol alone. This reviewer commends the authors for a well thought out study and offer the following recommendations to enhance the manuscript.

Many thanks for accepting the invitation to review our manuscript.

We appreciate the time you have spent reviewing our manuscript, and your expert comments and suggestions.

All changes to the manuscript are indicated with yellow highlighted text.

**Specific Comments:**

Introduction:

Line 72 add “and” before “increase plasma volume”.

Thank you for identifying this, this change has now been made.

Remove the double citation block on line 76. It is unclear if it should be (7, 17) or (11-16).

Many thanks for bringing this to our attention, this change has now been made.

Remove double citation block in line 80.

We again appreciate you for bringing this to our attention, this change has now been made.

You can separate the paragraphs for each hyperhydration agent.

Thank you for this suggestion. This paragraph has now been broken down into three separate paragraphs that focus on 1) water induced hyperhydration, 2) glycerol and sodium as hyperhydration agents, and 3) the introduction to combined ingestion and sodium bicarbonate as a source of sodium.

These updated paragraphs appear on lines 64-107 of the revised manuscript.

The introduction could benefit from a brief discussion of the potential drawbacks of NaHCO3 on gastrointestinal distress, thereby supporting your decision to include this measurement.

Thank you for raising this point. We have now added relevant information about the potential drawbacks of both NaHCO_3_ and glycerol supplementation. The following section has been added:

‘Additionally, following isolated ingestion of both glycerol and NaHCO_3_, GI symptoms have been reported (10, 21, 22). As additive incidence and severity of GI symptoms with the ingestion of combined glycerol and NaHCO_3_ is a possibility and may impact the ergogenic benefits of increased hydration status and/or buffering capacity (9, 23), it is essential to quantify these symptoms when investigating their combined ingestion.’

This text appears on lines 96-101 of the revised manuscript.

Methods

Line 195 appears redundant to line 175-176

Thank you for noticing this, we agree and have now removed what was line 195 in the originally submitted manuscript.

Line 205 – I recommend flipping the wording to make it clear that the subscript 0 refers to baseline, and subscript t refers to measurements at each subsequent timepoint, respectively.

Many thanks for this suggestion, we have now integrated this change at line 214-215 of the revised manuscript.

Lines 210-226 – You captured important information regarding menstrual cycle. Although you excluded days 1-3 of the menstrual cycle to minimize the influence of hormonal fluctuations on GI symptoms, was the ovulation test merely used to determine participants were not anovulatory? I am a little confused by the decision to only exclude this time rather than isolate to a specific phase, particularly when some of the authors of the study previously found a more pronounced increase in body mass in phase 4 of the menstrual cycle: <https://pubmed.ncbi.nlm.nih.gov/39591960/>

Thank you for your comment. Testing for the current project was conducted outside of days 1-3 of the menstrual cycling in an effort to avoid any influence of menses on reported GI symptoms. The decision to exclude days 1-3 of the menstrual cycle was based on data collected by our team (currently unpublished). To add more clarity surrounding this decision, additional references have been added to the revised manuscript which report increased severity and occurrence of gastrointestinal symptoms at the start of menstruation. These new references (42-44) can be found within the revised manuscript on line 225.

Given that testing in a specific menstrual cycle phase (or comparison between phases) was not the primary focus of this study, our study was designed using methodology consistent with a ‘silver’ tier in the recommendations published by Smith et al. (https://pubmed.ncbi.nlm.nih.gov/35168200/), however our team acknowledges in our ‘limitations and future research’ section that future research can aim to use ‘gold’ standard methodologies where feasible and when considering the primary aims of the study.

Line 246 – 250 – Rewrite this sentence for clarity; the second half can be omitted.

Thank you for this suggestion. This sentence has now been rewritten for clarity with parts of the second half of the sentence omitted. This sentence now reads as following:

‘For each GI symptom, the total point scores for severity were summed across all data collection time points and analysed using a Friedman’s two-way ANOVA by ranks test with a Bonferroni post hoc pairwise comparison in SPSS Statistics (Version 29; IBM Corporation, NY, USA) (48).’

This updated wording appears in lines 255-258 of the revised manuscript.

Results

Line 290 - why are both p>0.050 and the exact the p-values reported?

Thank you for bringing this to our attention. We have now removed the p>0.050 p value.

The authors spend majority of the results discussing the difference between each beverage and the control condition. But the main effect of interest is any difference between G+SB and SB or G alone. It may be worth explicitly stating these null differences between experimental conditions in the results section as well.

Thankyou for this suggestion. The null differences have now been explicitly stated. The statement consistently reads as following,

‘No significant differences were observed between G+SB, SB and G during the full 180 min post-ingestion trial period (p>0.0008)’

These changes appear at lines 282-284, 290-291, and 298-299 of the revised manuscript.

Discussion

Line 366 – I believe it is overstating your conclusions to say glycerol and sodium induced hydration does not increase body mass. Greater fluid retention is evidence of an increase. Instead, I think it is more accurate to say the magnitude of the typical daily body mass fluctuations within each person was more variable than the observed between conditions differences in body mass change across different participants. But I do not believe it is accurate to say the glycerol and sodium does not increase body mass. If you examine the relative changes in body mass rather than the absolute number, I believe your results would align with the fluid retention values.

Thank you for raising this point. We agree and have therefore removed the sentence you have referred to above.

Line 388-Good point about the time available before the race.

Thank you!

Line 445 – This appears to be the incorrect reference number.

This has now been updated along with some other minor errors that we noticed in the other references in this section. The cause of these minor inconsistencies was an error with the connection between the document and Endnote.
